# Dysregulation of apolipoprotein o reprograms CCR7^+^CD4^+^T cell fate in primary autoimmune thrombocytopenia

**DOI:** 10.1016/j.isci.2025.113792

**Published:** 2025-10-16

**Authors:** Tengda Li, Xiang Li, He Huang

**Affiliations:** 1 Key Laboratory of Laboratory Medicine, Ministry of Education, School of Laboratory Medicine and Life Sciences, Wenzhou Medical University, Wenzhou, Zhejiang 325035, China; 2 Department of Chemistry, Fudan University, Shanghai 200438, China; 3 Department of Hematology, The Second Affiliated Hospital and Yuying Children’s Hospital of Wenzhou Medical University, Wenzhou, Zhejiang, China

**Keywords:** immunology, hematology, transcriptomics

## Abstract

Primary immune thrombocytopenia involves antibody-driven platelet loss and perturbed CD4^+^ T cell regulation. By integrating single-cell transcriptomic, epigenetic, and functional analyses, we delineated CCR7^+^CD4^+^ T cell states with distinct metabolic and transcriptional programs. A subset enriched in patients exhibited reduced oxidative phosphorylation and enhanced glycolysis, accompanied by elevated expression of SP100 and its downstream transcriptional targets FOXP1 and CDK6. Trajectory analysis positioned these cells as developmentally arrested intermediates that, in normal individuals, mature into CCR7^+^ cells expressing apolipoprotein O (APOO). Functional perturbations revealed that APOO preserves oxidative metabolism and CCR7 identity while restraining SP100-dependent transcription. Methylation profiling identified APOO hypermethylation and transcriptional silencing in patient-derived CD4^+^ T cells. Together, these data define APOO as a metabolic-transcriptional checkpoint governing CCR7^+^CD4^+^ T cell fate, whose repression fosters dysfunctional differentiation and immune imbalance in autoimmune thrombocytopenia.

## Highlights

• ITP vs*.* Normal CD4^+^CCR7^+^T cells differ in transcription and metabolism
• APOO is altered at transcript and methylation levels in ITP CD4^+^T cells
• In ITP CD4^+^CCR7^+^T cells, APOO downregulation lowers CCR7 via OXPHOS and drives SP100
• APOO acts as a dual metabolic-transcriptional checkpoint and potential ITP target

## Graphical abstract

**Figure:**
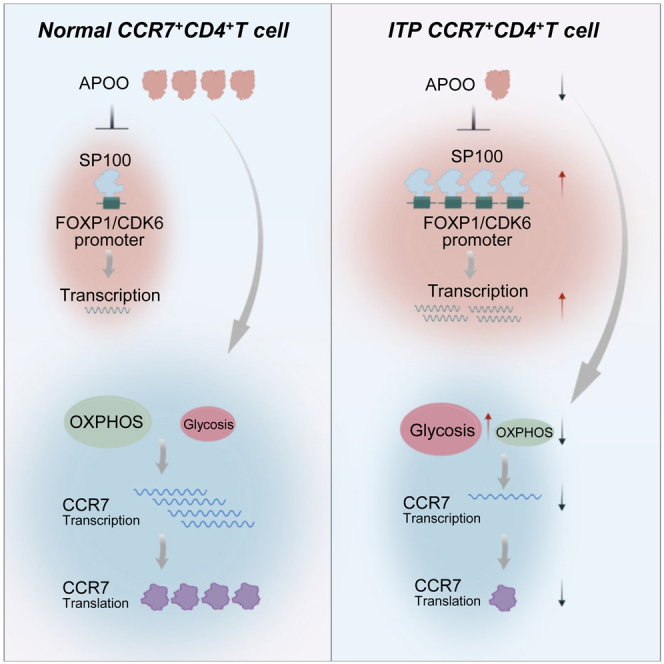

## Introduction

Primary immune thrombocytopenia (ITP) is an acquired autoimmune disorder characterized by isolated thrombocytopenia in the absence of other causes.^1^^,^^2^ The current diagnostic approach for ITP is largely exclusionary.^2^ In severe cases, profound thrombocytopenia may lead to spontaneous bleeding and necessitate invasive interventions.^2^ Although first-line treatments, such as corticosteroids, intravenous immunoglobulin (IVIG), and anti-D immunoglobulin are commonly employed, their efficacy is often transient.^2^ Patients who fail to respond require second-line therapies, including thrombopoietin receptor agonists and rituximab, while additional agents remain under clinical investigation.^2^ However, these treatments are frequently accompanied by adverse effects or limited durability, highlighting the need for safer and more effective therapeutic strategies.^2^^,^^3^ Fundamentally, the suboptimal outcomes in ITP management stem from an incomplete understanding of its pathogenesis.^2^^,^^3^ Accumulating evidence suggests that both humoral and cellular immune dysregulation contribute to enhanced platelet destruction and impaired megakaryopoiesis in patients with ITP.^2^

Among cellular players, CD4^+^T cells play a central role in orchestrating immune responses through cytokine secretion and interactions with other immune cells.^4^ In autoimmune conditions, CD4^+^T cell activation is frequently dysregulated.^5^ In ITP, aberrantly activated CD4^+^T cells have been shown to be increased and capable of recognizing platelet antigens, thereby contributing to the generation of pathogenic autoantibodies.^5^ Functional or compositional imbalances among CD4^+^T cell subsets have been implicated as pivotal drivers of disease onset and progression.^6^ Pharmacological suppression of T cell responses—such as with dexamethasone—has been demonstrated to inhibit CD4^+^T cell proliferation and reduce cytotoxic T lymphocyte-mediated platelet lysis, further supporting the importance of T cell-targeted regulation.^7^ Moreover, recent studies have identified metabolic reprogramming in CD4^+^T cells as a therapeutic avenue in ITP, as exemplified by the effect of empagliflozin in modulating T cell metabolism.^8^ These findings collectively underscore the essential role of CD4^+^T cells in the pathophysiology of ITP and the potential of targeting their functional abnormalities for therapeutic benefit.

C-C chemokine receptor type 7 (CCR7), a chemokine receptor involved in the homing of T cells and dendritic cells to secondary lymphoid organs, has emerged as a critical marker in autoimmune research.^9^ Low expression of CCR7 (CCR7^low^) is associated with activated T follicular helper cells (Tfh) and linked to ongoing autoreactive antibody production in several autoimmune diseases.^10^ In CCR7-deficient mice, spontaneous autoimmune gastritis and ectopic lymphoid structure formation have been observed, along with the accumulation of activated T cells in mucosal and lymphoid tissues.^11^ In the context of ITP, increased frequencies of CCR7^+^CD4^+^cells have been reported in newly diagnosed and relapsed patients, suggesting a potential role for CCR7-expressing CD4^+^T cell in disease activity and recurrence.^12^ Nevertheless, the internal heterogeneity of the CD4^+^CCR7^+^T cell population in ITP remains poorly understood, as most studies have treated this group as a homogeneous entity. Consequently, standardized criteria for their quantification and characterization are lacking.

In this study, we employed single-cell RNA sequencing to profile peripheral blood CD4^+^T cells from normal individuals and ITP patients. We delineated distinct transcriptional subpopulations and identified several subsets exhibiting marked alterations in ITP. Notably, we focused on the CCR7^+^CD4^+^T cell compartment and uncovered previously unrecognized heterogeneity within this population. Our findings revealed functionally divergent subclusters. Furthermore, we identified key molecular drivers selectively enriched in disease-associated CCR7^+^subsets. These results provide novel mechanistic insights into the contribution of CD4^+^T cell subpopulations to ITP pathogenesis and offer potential targets for future therapeutic development.

## Results

### Distinct global features of CD4^+^T cell subsets in ITP patients

**Figure 1:**
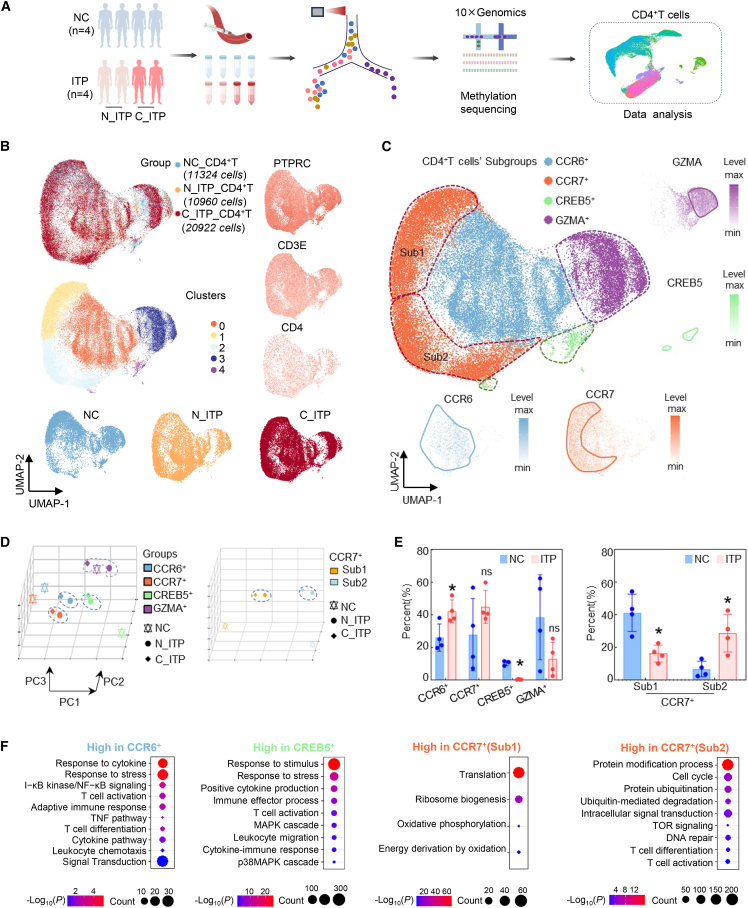
Overall characterization of CD4^+^T cells from primary immune thrombocytopenia (ITP) patients and controls at the single-cell level (A) Workflow for sample collection, sequencing and downstream analysis. (B) Integration of all samples, with visualization of cells by origin and cluster, and expression levels of CD4, CD3E, and PTPRC. (C) Results of cell-type annotation. (D) Comparison of principal component features across cell populations in different groups. (E) Statistical analysis of the proportions of each T cell subset in ITP versus normal control (NC) groups, data are presented as mean ± standard deviation. (F) Pathway enrichment analysis of genes upregulated within each subset. NC, normal control; N_ITP, newly diagnosed ITP patients; C_ITP, chronic ITP patients; ITP, included both N_ITP and C_ITP. ∗*p* < 0.05; ns, not significant.

To investigate CD4^+^T cell heterogeneity in ITP, we performed single-cell RNA sequencing and DNA methylation profiling on peripheral CD4^+^T cells from four normal donors and four ITP patients (two newly diagnosed, two chronic) (Figure 1A). Unsupervised clustering identified five transcriptionally distinct subsets (clusters 0–4). Cluster 1 was expanded and cluster 2 reduced in normal controls (NC), whereas the opposite pattern was observed in ITP samples (Figure 1B). Based on canonical markers, these were annotated as C-C chemokine receptor type 6 (CCR6)^+^, CCR7^+^, cAMP responsive element binding protein 5 (CREB5)^+^, and granzyme A (GZMA)^+^subsets (Figure 1C). The CCR7^+^population comprised two transcriptionally distinct subgroups: CCR7^+^(Sub1, cluster 1) and CCR7^+^(Sub2, cluster 2), enriched in normal and ITP samples, respectively (Figures 1B and 1C).

**Figure 2:**
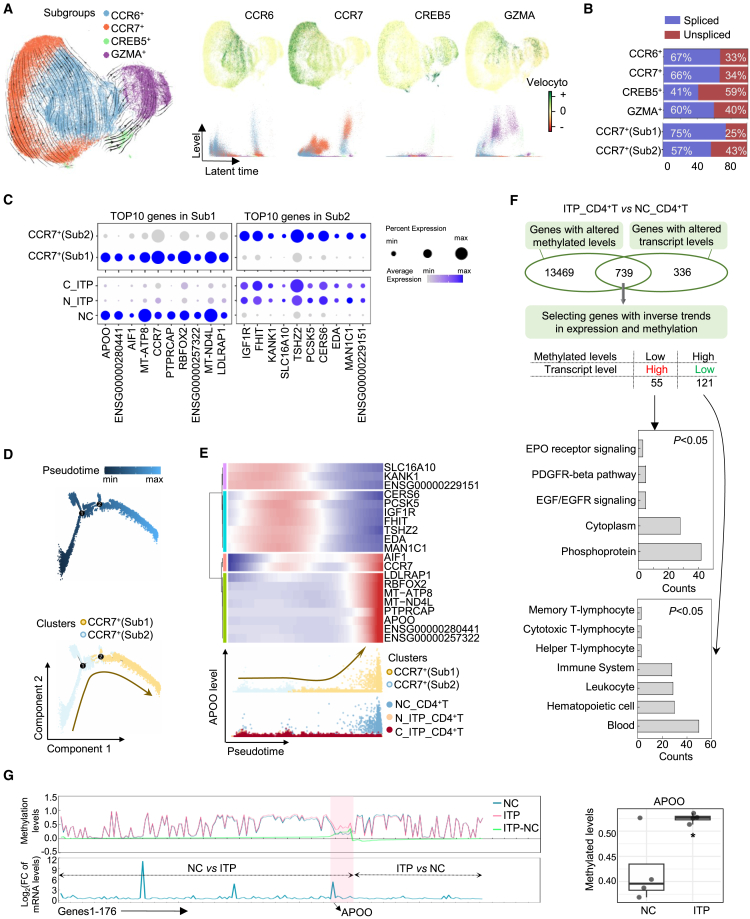
Characteristic gene profiling in the CCR7^+^ (Sub1 and Sub2) subpopulations (A) Transcriptional dynamics of CCR6, CCR7, and other relevant genes over pseudotime in different T cell subsets, as inferred by scVelo analysis, including RNA velocity patterns across clusters. (B) Spliced and unspliced RNA ratios across T cell subsets. (C) Differential expression of the top 10 most variable genes in CCR7^+^ (Sub1 and Sub2) clusters across different sample origins. (D and E) Pseudotime trajectory analysis of CCR7^+^(Sub1 and Sub2) subsets (D), along with dynamic expression patterns of their respective top 10 marker genes over pseudotime (E). (F) Integration of CD4^+^T cell methylation sequencing data from ITP patients and NC with single-cell transcriptomic data to identify consistently regulated genes and perform pathway enrichment analysis. (G) Joint visualization of methylation and mRNA expression levels for 176 genes exhibiting concordant changes in both datasets, highlighting APOO as the gene with the most pronounced variation and presenting its methylation profile. The boxplot primarily illustrates the median, quartiles. (H) Pseudotemporal transcriptional trajectory of APOO, along with its expression levels across different groups. NC, normal control; N_ITP, newly diagnosed ITP patients; C_ITP, chronic ITP patients; ∗*p* < 0.05.

Principal-component analysis (PCA) indicated that transcriptomic features of CD4^+^T cell subsets from newly diagnosed ITP (N_ITP) and chronic ITP (C_ITP) were broadly similar, with relatively small separations between these two categories, whereas both were mostly separated from NC (Figure 1D, left). Moreover, the CCR7^+^(Sub1) and CCR7^+^(Sub2) populations showed pronounced differences between ITP (including both N_ITP and C_ITP) and NC, while differences between N_ITP and C_ITP within these subsets were comparatively modest (Figure 1D, right). Proportional analysis showed increased CCR6^+^ or CCR7^+^(Sub2) and decreased CREB5^+^ in ITP, with a reduction of CCR7^+^(Sub1) (Figure 1E). scVelo analysis indicated elevated transcriptional potential across CCR6^+^, CCR7^+^, CREB5^+^, and GZMA^+^subsets, with higher unspliced transcript levels in CREB5^+^ and CCR7^+^(Sub2), suggesting enhanced transcriptional activity or plasticity (Figures 2A and 2B).

Functionally, CD4^+^T cells from normal donors were enriched in oxidative phosphorylation (OXPHOS), T cell activation, and immune response, while N_ITP samples showed upregulation of T cell receptor signaling, interferon signaling, and response to stimulus; C_ITP cells were enriched in peptide chain elongation, SRP-dependent protein targeting or GCN2 amino acid response (Figure S1). Subset-level analysis showed distinct pathway enrichments: CCR6^+^ in cytokine/stress response, CREB5^+^ in stimulus response, CCR7^+^ in T cell activation, and GZMA^+^ in immune effector process (Figures 1F and S1). CCR7^+^(Sub1) and CCR7^+^(Sub2) displayed divergent enrichment patterns, reflecting functional bifurcation (Figure 1F). Together, these findings highlighted a shift in CD4^+^T cell programs in ITP from immune homeostasis toward inflammation and stress adaptation.

### CCR7^+^(Sub1) and CCR7^+^(Sub2) were distinct subsets, with CCR7^+^(Sub2) being less mature and enriched in ITP. APOO downregulation was likely linked to the immaturity of CCR7^+^(Sub2) cells

Traditionally, CCR7^+^CD4^+^T cells were regarded as a single population, overlooking their heterogeneity. scRNA-seq revealed two transcriptionally distinct subsets: CCR7^+^(Sub1) and CCR7^+^(Sub2). CCR7^+^(Sub1) highly expressed genes related to translation, ribosome biogenesis, and OXPHOS, while CCR7^+^(Sub2) upregulated genes involved in protein modification process, protein ubiquitination, and cell cycle (Figure 1F). scVelo analysis revealed that CCR7 expression increased at intermediate stages along the differentiation trajectory and reached a pronounced peak at the terminal stage, significantly exceeding earlier expression levels (Figure 2A). In addition, a higher proportion of unspliced transcripts were observed in the Sub2 population, suggesting a less mature cellular state (Figure 2B).

Apolipoprotein O (APOO), a top marker of Sub1, was nearly absent in Sub2 and markedly downregulated in ITP-derived CD4^+^T cells (Figure 2C). Conversely, Sub2 markers were upregulated in ITP (Figure 2C). No overlap was observed between the top 10 markers of the two subsets (Figure 2C). Monocle analysis positioned Sub2 at the early stage of differentiation and Sub1 at the terminal state (Figure 2D). APOO expression increased along pseudotime, peaking at the terminal phase (Figure 2E). CCR7 expression showed a late-stage peak that coincided with APOO elevation, they were low in immature CCR7^+^(Sub2) or ITP-derived CD4^+^T cells (Figures 2E and 2H). These results suggested that in ITP, CCR7^+^T cells were arrested in an immature Sub2-like state probably due to low APOO expression.

To further confirm our hypothesis, we integrated transcriptomic and methylation data from CD4^+^T cells of ITP patients and NC. Due to the suppressive effect of DNA methylation on gene expression, we selected genes exhibiting inverse patterns between methylation and transcriptional levels (a total of 176 genes, Figure 2F). Among them, 55 genes showed increased transcription and decreased methylation in CD4^+^T cells from ITP patients, whereas 121 genes exhibited reduced transcription and increased methylation (Figure 2F). Pathway enrichment analysis of these genes revealed that the 55 upregulated genes in ITP patients were primarily enriched in the EPO receptor signaling, PDGFR-beta pathway, EGF/EGFR signaling pathway (Figure 2F). In contrast, the genes upregulated in CD4^+^T cells from NC were mainly enriched in pathways related to memory T-lymphocyte, cytotoxic T-lymphocyte, helper T-lymphocyte (Figure 2F). Among them, APOO showed the most pronounced inverse correlation—hypomethylated and highly expressed in NC but hypermethylated and suppressed in ITP (Figures 2F and 2G). These results indicate in ITP, the aberrant downregulation of APOO in CD4^+^T cells may hinder the differentiation of CCR7^+^(Sub2) cells into terminal CCR7^+^(Sub1) cells, potentially contributing to the reduced proportion of the CCR7^+^(Sub1) subset.

### SP100 and its downstream targets FOXP1 and CDK6 were enriched in CCR7^+^(Sub2) and inversely correlated with APOO

**Figure 3:**
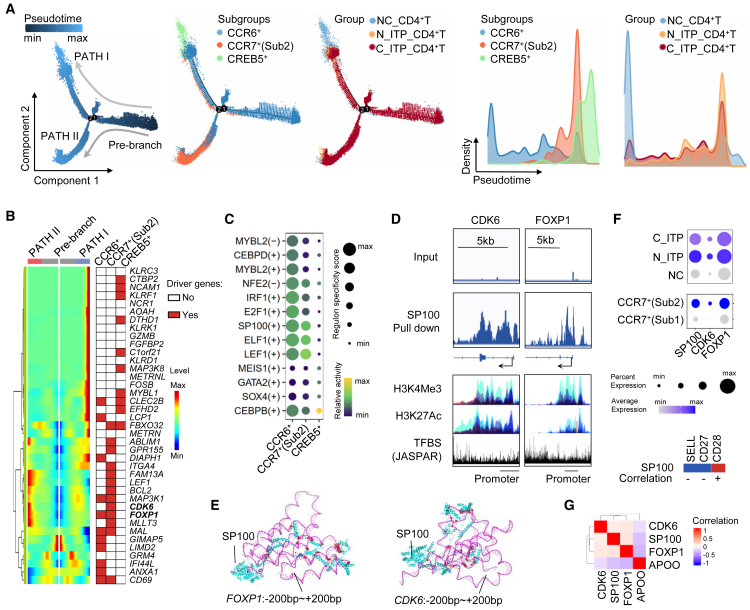
Differentiation trajectory analysis of altered CD4^+^T cell subsets in ITP and identification of key regulatory genes in the CCR7^+^(Sub2) population (A) Pseudotime trajectory analysis of CCR6^+^, CCR7^+^(Sub2), and CREB5^+^T cell subsets. (B) Visualization of representative driver genes associated with distinct differentiation pathways, highlighting key regulators in CCR6^+^, CCR7^+^(Sub2), and CREB5^+^subsets. (C) Inference of transcriptional regulon activity across different T cell subsets. (D) ChIP-seq analysis of the transcription factor SP100, examining its binding to CDK6 and FOXP1 promoter regions and associated genomic features such as histone modifications and transcription factor binding motifs. (E) Molecular docking analysis of SP100 with the promoter regions of CDK6 and FOXP1, including visualization of protein–DNA interface and hydrogen bond interactions. (F) Expression levels of SP100 and related genes across distinct cell populations, along with correlation analyses between SP100 and other genes. (G) Correlation analysis among SP100, CDK6, APOO, and FOXP1 at the single-cell level. NC, normal control; N_ITP, newly diagnosed ITP patients; C_ITP, chronic ITP patients.

We analyzed transcriptional features of cell clusters showing significant shifts in ITP, including CCR6^+^, CCR7^+^(Sub2), and CREB5^+^subsets. Pseudotime trajectory analysis showed that CCR6^+^cells were positioned at the early stage of differentiation, whereas CCR7^+^(Sub2) and CREB5^+^cells were located at later stages (Figure 3A). Most ITP-derived cells were enriched in the CCR7^+^(Sub2) state, consistent with earlier observations (Figure 3A). We then examined differentiation-associated genes between CCR6^+^cells and their downstream lineages. CCR6^+^cells transitioning into CCR7^+^(Sub2) or CREB5^+^subsets exhibited distinct sets of upregulated genes (Figure 3B). Integration of these genes with velocity-derived driver genes revealed critical subset-specific factors such as: LIM domain containing 2 (LIMD2) in CCR6^+^, forkhead box P1 (FOXP1), and cyclin-dependent kinase 6 (CDK6) in CCR7^+^(Sub2), and mitogen-activated protein kinase kinase kinase 8 (MAP3K8) in CREB5^+^cells, suggesting their essential roles in lineage maintenance (Figure 3B).

Regulon analysis identified transcription factors with high activity in CCR7^+^(Sub2), including SP100 nuclear antigen (SP100) (+) and lymphoid enhancer-binding factor 1 (LEF1) (+) (Figure 3C). Chromatin immunoprecipitation followed by sequencing (ChIP-seq) analysis demonstrated that among the candidate transcription factors, only SP100 showed significant binding to the promoter regions of FOXP1 and CDK6 (Figure 3D). These binding regions coincided with H3K4Me3 and H3K27Ac enrichment, as well as strong transcription factor binding signals, suggesting regulatory activity at these loci (Figure 3D). Using AlphaFold3, we modeled the interactions between SP100 and the promoter regions (−200 to +200 bp) of FOXP1 and CDK6. The presence of extensive hydrogen bonding indicated strong binding affinity (Figure 3E).

Expression analysis confirmed that SP100, FOXP1, and CDK6 were upregulated in both N_ITP and C_ITP samples and were particularly elevated in the CCR7^+^(Sub2) subset (Figure 3F). We further found SP100 expression negatively correlated with SELL and CD27, but positively with CD28, which may explain the limited CCR7 expression levels in SP100-high CCR7^+^(Sub2) cells (Figures 3F and 2E). Moreover, the SP100–FOXP1–CDK6 regulon was negatively correlated with APOO (Figure 3G). Given the reduced APOO expression in CCR7^+^(Sub2) cells from ITP patients, this downregulation may contribute to SP100 upregulation and subsequent activation of FOXP1 and CDK6. These findings suggest that APOO deficiency in ITP may drive the expansion of a dysfunctional CCR7^+^CD4^+^T cell subset with impaired CCR7 expression due to aberrant SP100-mediated regulation.

### APOO downregulation in CD4^+^CCR7^+^T cells of ITP patients impaired OXPHOS, suppressed CCR7, and induced SP100 expression

**Figure 4:**
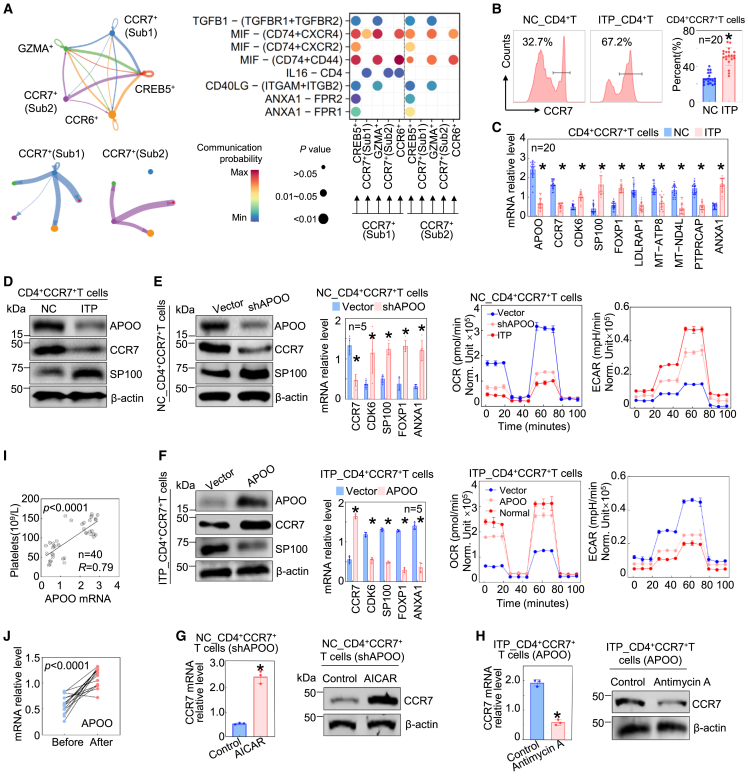
The regulatory role of APOO in CD4^+^CCR7^+^T cells (A) Inferred intercellular communication among different T cell subsets, and major signaling pathways through which CCR7^+^(Sub1/2) subsets act as sender cells to communicate with other cell populations. (B and C) Comparison of the proportion of CD4^+^CCR7^+^T cells between NC and ITP patients (B), and differential expression of key genes across groups (C). (D) Protein levels of APOO, CCR7, SP100 in CD4^+^CCR7^+^T cells from normal individuals and ITP patients. (E) APOO knockdown in CD4^+^CCR7^+^T cells derived from the normal and protein levels of CCR7, SP100 after APOO knockdown, and assessment of resulting changes in the expression of critical genes, and seahorse analysis comparing CD4^+^CCR7^+^T cells from ITP patients and those from NC with or without APOO knockdown. (F) In CD4^+^CCR7^+^T cells derived from ITP patients, APOO was overexpressed and the changes in key molecules at both protein and transcriptional levels were examined. The OXPHOS and glycolytic capacities were then compared with those of normal control cells. (G) In APOO knockdown-normal CD4^+^CCR7^+^T cells, AICAR was added, after which CCR7 expression was evaluated at both transcriptional and protein levels. (H) In CD4^+^CCR7^+^T cells from ITP patients, APOO was overexpressed followed by Antimycin A treatment, and the transcriptional and protein levels of CCR7 were assessed. (I) The correlation between APOO transcriptional levels and platelet counts was analyzed. (J) The changes in APOO transcriptional levels before and after treatment were determined. In the statistical plots shown in (B–C and E–F and G–H), data are presented as mean ± standard deviation. NC, normal control; ITP, included both N_ITP and C_ITP; ∗*p* < 0.05.

To explore the intercellular communication characteristics between CCR7^+^(Sub1), CCR7^+^(Sub2), and other CD4^+^T cell subsets, we conducted CellChat analysis. Both CCR7^+^subsets exhibited strong interactions with other CD4^+^T cell populations (Figure 4A). The key signaling pathways identified in CCR7^+^(Sub1) were also active in CCR7^+^(Sub2), with comparable pathway intensities (Figure 4A). Notably, annexin A1 (ANXA1)-mediated signaling activity was elevated in CCR7^+^(Sub2) cells (Figure 4A).

We next analyzed peripheral blood samples from 20 normal donors and 20 ITP patients. Flow cytometry revealed a higher proportion of CD4^+^CCR7^+^T cells in the ITP group (Figure 4B). Gene expression analysis showed that APOO, CCR7, and protein tyrosine phosphatase, receptor type C-associated protein (PTPRCAP) were enriched in CD4^+^CCR7^+^T cells from normal individuals, while SP100, FOXP1, ANXA1, and CDK6 were upregulated in ITP-derived cells (Figure 4C). SP100 protein levels were elevated in CD4^+^CCR7^+^T cells from ITP patients, whereas APOO and CCR7 protein levels were higher in NC (Figure 4D).

To investigate the regulatory role of APOO, we knocked down APOO in CD4^+^CCR7^+^T cells from NC (Figure 4E). Silencing of APOO led to a reduction in CCR7 expression and an upregulation of SP100 expression (Figure 4E). Furthermore, APOO knockdown resulted in increased mRNA levels of SP100, FOXP1, CDK6, and ANXA1, alongside a decreased mRNA level of CCR7 (Figure 4E), while the expression of other genes remained largely unaffected (data not shown). Seahorse analysis revealed that APOO knockdown reduced OXPHOS capacity, as evidenced by diminished maximal respiration, ATP production, and basal respiration (Figure 4E). However, the extent of decline was less than that observed in ITP cells (Figure 4E). Compensatory glycolysis was increased but to a lesser extent than in the ITP group (Figure 4E). We performed APOO overexpression in CD4^+^CCR7^+^T cells derived from ITP patients. As shown in Figure 4F, APOO overexpression increased CCR7 expression and reduced SP100 expression. At the transcriptional level, APOO overexpression led to elevated CCR7 while reducing CDK6, SP100, FOXP1, and ANXA1 levels in ITP patient-derived CD4^+^CCR7^+^T cells, consistent with our expectations (Figure 4F). Furthermore, APOO overexpression enhanced OXPHOS while reducing glycolysis in these cells, bringing their metabolic phenotype closer to that of NC (Figure 4F). These findings demonstrate that in CD4^+^CCR7^+^T cells, APOO positively regulates CCR7 while suppressing SP100, thereby influencing downstream transcriptional programs. Moreover, APOO was essential for maintaining OXPHOS in CD4^+^CCR7^+^T cells. In ITP, APOO deficiency might impair CCR7 expression and mitochondrial respiration, contributing to a shift toward glycolytic metabolism and functional dysregulation in this T cell subset.

To determine whether APOO influences CCR7 transcription and expression through metabolic regulation, we treated APOO-knockdown normal CD4^+^CCR7^+^T cells with the AMP-activated protein kinase (AMPK) agonist 5-aminoimidazole-4-carboxamide ribonucleotide (AICAR), and APOO-overexpressing ITP patient–derived CD4^+^CCR7^+^T cells with the mitochondrial complex III inhibitor antimycin A. As shown in Figures 4G and 4H, CCR7 transcription and protein levels were restored in APOO-knockdown normal CD4^+^CCR7^+^T cells upon AICAR treatment, whereas CCR7 transcription and protein levels were reduced in APOO-overexpressing ITP CD4^+^CCR7^+^T cells following antimycin A treatment. These results indicate that enhancing or inhibiting cellular energy metabolism with AICAR or antimycin A, respectively, can rescue the regulatory effects of APOO knockdown or overexpression on CCR7, suggesting that APOO modulates CCR7 transcription and thereby protein expression through metabolic pathways.

Moreover, APOO mRNA levels showed a positive correlation with individual platelet counts (Figure 4I). In addition, our results demonstrated that the transcriptional level of APOO was significantly increased after treatment, with the difference reaching statistical significance (Figure 4J). Taken together, these findings support the potential of APOO as a sensitive biomarker for therapeutic response in ITP.

## Discussion

ITP is an autoimmune hematological disorder characterized by thrombocytopenia that may lead to abnormal bleeding, including potentially life-threatening hemorrhages in vital organs.^1^^,^^2^ Immune dysregulation plays a central role in disease pathogenesis, and accumulating evidence suggests that CD4^+^T cells are critical mediators in the development of ITP.^5^ In this study, we performed single-cell RNA sequencing of CD4^+^T cells from ITP patients and NC, enabling us to resolve multiple transcriptionally distinct subsets.

CCR7, a chemokine receptor essential for CD4^+^ T cell homing to lymphoid tissues, plays a critical role in maintaining immune homeostasis and initiating primary immune responses.^9^ Traditionally, CD4^+^CCR7^+^T cells have been studied as a uniform population, overlooking their potential heterogeneity. This simplification may partly explain why the true functional properties and proportions of CD4^+^CCR7^+^T cells in ITP remain poorly understood. Recent studies have highlighted the diverse roles of CCR7 across autoimmune diseases. For example, a CCR7^low^ Treg subset has been implicated in dysfunctional interferon responses in systemic lupus erythematosus (SLE)^13^; αβ T cells lacking CCR7 are enriched in coronary plaques^14^; and CCR7 expression in peripheral CD4^+^T cells has been linked to disease activity in rheumatoid arthritis and active SLE.^15^^,^^16^ These findings suggest that even within CD4^+^CCR7^+^T cells, functional heterogeneity may exist due to intrinsic differences in gene expression among subpopulations. In our study, we subdivided the CD4^+^CCR7^+^T cell population into two distinct subsets: CCR7^+^(Sub1) and CCR7^+^(Sub2) (Figure 1C). These subsets exhibited divergent features. Sub1 cells (high CCR7) were enriched in normal individuals, while Sub2 cells (low CCR7) were expanded in ITP patients (Figures 1E and 2E). Genes highly expressed in CCR7^+^(Sub1) were strongly upregulated in normal individuals but were nearly undetectable in CCR7^+^(Sub2) and ITP-derived CD4^+^T cells (Figure 2C).

APOO is a protein localized to the inner mitochondrial membrane, primarily involved in the regulation of mitochondrial function and energy metabolism.^17^^,^^18^^,^^19^ Studies have shown that APOO inactivation suppresses thermogenesis in brown adipose tissue by reducing mitochondrial long-chain fatty acid oxidation.^17^ APOO has also been proposed as a metabolic regulator of systemic cholesterol homeostasis and a potential therapeutic target for atherosclerosis management.^18^ In macrophages, APOO deficiency alleviates advanced atherosclerosis and necrotic core expansion by promoting enhanced efferocytosis.^19^ However, the role of APOO in CD4^+^ T cells and autoimmune diseases has not been previously reported. Interestingly, we observed that APOO expression gradually increased as CCR7^+^(Sub2) cells differentiated toward the CCR7^+^(Sub1) phenotype (Figures 2E and 2H). Furthermore, APOO was significantly upregulated at the transcript level and exhibited reduced DNA methylation in CD4^+^T cells from normal individuals (Figures 2E–2G).

SP100 is a nuclear body-associated protein involved in transcriptional regulation, chromatin remodeling, and antiviral immunity.^20^^,^^21^^,^^22^^,^^23^ It has been identified as a major autoantigen in conditions such as hyper-IgM syndrome and primary biliary cholangitis (PBC).^20^^,^^21^^,^^22^ FOXP1, a forkhead family transcription factor, regulates immune cell development and is a known negative regulator of Tfh differentiation and interleukin-9 production in CD4^+^T cells.^24^^,^^25^^,^^26^ CDK6 is a nuclear and cytoplasmic cyclin-dependent kinase involved in G1-S cell cycle progression and cellular proliferation.^27^ ANXA1 is a calcium-dependent phospholipid-binding protein implicated in inflammation, apoptosis, and immune regulation, and can be secreted via non-classical pathways to exert anti-inflammatory effects.^28^^,^^29^ In CCR7^+^(Sub2) and ITP-derived CD4^+^ T cells, SP100, FOXP1, CDK6, and ANXA1 signaling were upregulated (Figures 3 and 4A). APOO and CCR7 were highly expressed in CD4^+^CCR7^+^T cells from NC, while SP100-related genes were elevated in ITP (Figures 4C and 4D). APOO mRNA positively correlated with platelets’ counts, and it promoted CCR7 expression and OXPHOS, while suppressing SP100 protein and the transcription of FOXP1 and CDK6 (Figure 4E, 4F, and 4I). These findings suggest that APOO deficiency in ITP impairs CCR7^+^T cell metabolism and enhances SP100-driven regulatory programs. Despite their increased proportion, CCR7^+^CD4^+^T cells in ITP may be functionally compromised due to reduced APOO activity.

In conclusion, our study identified two transcriptionally distinct CCR7^+^CD4^+^T cell subsets: CCR7^+^(Sub2), characterized by low CCR7 expression and enriched in ITP, and CCR7^+^(Sub1), marked by high CCR7 expression and predominant in normal individuals. APOO was shown to increase CCR7 expression by enhancing OXPHOS, while suppressing SP100-mediated signaling. The APOO deficiency observed in ITP may contribute to the dysfunctional phenotype of CCR7^+^CD4^+^T cells in this disease. Restoring APOO activity to recover CCR7^+^T cell function represents a potential therapeutic strategy for ITP and warrants further investigation.

### Limitations of the study

This study has certain limitations. The role of APOO as a sensitive biomarker for therapeutic responsiveness requires further validation in larger cohorts of clinical samples. Moreover, the key mechanisms by which APOO regulates CCR7 and SP100 proteins in CCR7^+^CD4^+^T cells need to be confirmed through expanded clinical investigations. In addition, the potential of APOO as a therapeutic target should be validated, and its precise molecular mechanisms remain to be elucidated. These questions will constitute important directions for our future research.

## Resource availability

### Lead contact

Further information and requests for resources and data should be directed to and will be fulfilled by the lead contact, Tengda Li (tengdali@wmu.edu.cn).

### Materials availability

This study did not generate any new unique reagents.

### Data and code availability

The single-cell RNA sequencing data of CD4^+^ T cells from patients with ITP have been deposited in Zenodo at https://zenodo.org/records/16941253.This study did not generate any new code. Any additional information required to reanalyze the data reported in this paper is available from the lead contact upon reasonable request.

## Acknowledgments

This research was supported by 10.13039/501100004731Zhejiang Provincial Natural Science Foundation of China under grant no. LQ24H100005, National Natural Science Foundation of China (NSFC) under grant no. 82503998, Wenzhou Basic Public Welfare Research project no. 2023Y0784/Y2023091, Talent Research Startup Project of 10.13039/100007835Wenzhou Medical University no.QTJ21020, Scientific research business expenses of Wenzhou Medical University no. KYYW202318.

## Author contributions

T.L., X.L. were responsible for writing the R code, data analysis, T.L. draft the manuscript. T.L. and X.L. conducted the experiments, while H.H. provided the clinical samples. T.L. revised and edited the manuscript. All authors have reviewed and approved the final version of the manuscript and take full responsibility for its content.

## Declaration of interests

The authors declare that they have no conflicts of interest.

## STAR★Methods

### Key resources table

**Table:** 

REAGENT or RESOURCE	SOURCE	IDENTIFIER
**Antibodies**		

PE/Cyanine7 anti-human CD4 Antibody	Biolegend	Cat#300511; RRID: AB_314079
APC/Cyanine7 anti-human CD8 Antibody	BioLegend	Cat# 344713; RRID: AB_2044005
APC anti-human CD45 Antibody	BioLegend	Cat# 304011; RRID: AB_314399
FITC anti-human CD197 (CCR7) Antibody	BioLegend	Cat# 353215; RRID: AB_10945291
Rabbit anti-human Apolipoprotein O	Thermo Fisher Scientific	Cat#PA5-145414; RRID: AB_3093622
Rabbit anti-human CCR7 antibody	Abcam	Cat# ab32075; RRID: AB_726207
Rabbit anti-human SP100 antibody	Proteintech	Cat# 84012-5-RR; RRID: AB_3671580
Rabbit anti-human β-actin antibody	Abcam	Cat# ab8227; RRID: AB_2305186
Goat Anti-Rabbit IgG H&L (HRP)	Abcam	Cat# ab205718; RRID: AB_2819160
Monoclonal anti-human CD3 and CD28 antibodies covalently bound to DynaBeads magnetic Beads	Thermo Fisher Scientific	Cat# 11131D; RRID: AB_2943359

**Chemicals, peptides, and recombinant proteins**		

Human IL-2 Recombinant Protein	Thermo Fisher Scientific	PHC0021
Antimycin A	Sigma	1397-94-0
AICAR	Sigma	2627-69-2

**Critical commercial assays**		

Seahorse XF Cell Mito Stress Test Kit	Agilent	103010–100
Seahorse XF Glycolysis Stress Test Kit	Agilent	103017–100

**Deposited data**		

Single-cell RNA sequencing of normal CD4^+^T cell (public data)	Luo Y et al.^30^	GEO: GSE175604(https://www.ncbi.nlm.nih.gov/geo/query/acc.cgi?acc=GSE175604)
Single-cell RNA sequencing of peripheral blood mononuclear cells (public data)	Zhao J et al.^31^	GEO: GSE255566(https://www.ncbi.nlm.nih.gov/geo/query/acc.cgi?acc=GSE255566)
SP100-ChIP-seq data (public data)	Arttu Jolma et al.^32^	GEO: GSM8590383(https://www.ncbi.nlm.nih.gov/geo/query/acc.cgi?acc=GSM8590383)
DNA methylation data of CD4^+^T cell from the normal and ITP patients	Du H et al.^33^	GEO: GSE205495(https://www.ncbi.nlm.nih.gov/geo/query/acc.cgi?acc=GSE205495)
Single-cell RNA sequencing of CD4^+^T cells of ITP	This paper	Zenodo: https://zenodo.org/records/16941253

**Oligonucleotides**		

Primers for RT-PCR, see Table S2	This paper	N/A

**Recombinant DNA**		

GIPZ Lentiviral Human APOO shRNA	Horizon	Cat# RHS4430-200167255; RRID: N/A
pLenti-EF1α-IRES-Puro	Addgene	Cat# 85132; RRID: Addgene_85132

**Software and algorithms**		

R	R Core Team, The R Foundation for Statistical Computing (CRAN)	RRID: SCR_001905; version 4.4.1; https://www.r-project.org/
Prism (GraphPad Prism)	GraphPad Software, LLC (Dotmatics)	RRID: SCR_002798; version 8.4.3; https://www.graphpad.com
Integrative Genomics Viewer (IGV)	James T.R. et al.^34^	RRID: SCR_011793; version 2.14.1; https://igv.org
AlphaFold3 server	Google DeepMind & Isomorphic Labs	RRID: SCR_025885; accessed 2025-02-10; https://alphafoldserver.com
PyMOL	Schrodinger, LLC	RRID: SCR_000305; version 3.1.3; https://pymol.org
FlowJo™	FlowJo, LLC (BD Life Sciences)	RRID: SCR_008520; version v10.10; https://www.flowjo.com/solutions/flowjo
BioGDP	Jiang, S. et al.^35^	RRID: N/A; version N/A; https://biogdp.com/
Python	Python Software Foundation	RRID: SCR_008394; version 3.7.12; https://www.python.org
STRING	STRING Consortium	RRID: SCR_005223; version 12.0; https://string-db.org
CellRanger	10x Genomics	RRID: SCR_017344; version 8.0; https://www.10xgenomics.com/support/software/cell-ranger
scVelo	Volker Bergen et al.^36^	RRID: SCR_018168; version 0.3.3; https://github.com/theislab/scvelo
pySCENIC	Bram Van de Sande et al.^37^	RRID: SCR_025802; version 0.12.1; https://pyscenic.readthedocs.io
CellChat	Suoqin Jin et al.^38^	RRID: SCR_021946; version 2.1.0; https://github.com/sqjin/CellChat
ComplexHeatmap	Zuguan Gu et al.^39^; Zuguan Gu.^40^	RRID: SCR_017270; version 2.24.1; https://bioconductor.org/packages/ComplexHeatmap
Seurat	Yuhan Hao et al.^41^	RRID: SCR_016341; version v5; https://satijalab.org/seurat/
Monocle	Trapnell C et al.^42^; Xiaojie Qiu et al.^43^; Xiaojie Qiu et al.^44^	RRID: SCR_016339; version v2.36.0; https://bioconductor.org/packages/monocle

### Experimental model and study participant details

#### Human subjects

The diagnosis of ITP was based on clinical exclusion. In addition to detailed history-taking and physical examination, the diagnostic criteria included: (1) a consistently reduced platelet count confirmed by at least two separate complete blood count tests, with no significant morphological abnormalities observed in peripheral blood smears; (2) absence of splenomegaly; (3) bone marrow examination showing normal or increased megakaryocytes with maturation arrest; (4) exclusion of other causes of secondary thrombocytopenia; (5) measurement of serum thrombopoietin (TPO) levels, which assists in distinguishing ITP (normal TPO) from bone marrow failure syndromes (elevated TPO); and (6) detection of platelet glycoprotein-specific autoantibodies, which help differentiate immune from non-immune causes. Newly diagnosed ITP refers to cases within 3 months of diagnosis, and chronic ITP refers to cases with persistent thrombocytopenia for over 12 months. The control group comprised individuals with normal hematologic profiles.

Peripheral blood samples were collected from 20 NC (*n* = 20) and 24 ITP patients (*n* = 24), including 12 N_ITP and 12 C_ITP cases. Samples were allocated to experimental groups based on disease stage. Among these, 2 N_ITP and 2 C_ITP samples were used for single-cell RNA sequencing (scRNA-seq), while the remaining samples were used for validation experiments. The average ages of the NC, N_ITP, and C_ITP groups were 52.1, 51.2, and 51.8 years, respectively, with a male-to-female ratio of 1:1 in each group. Among patients with ITP, platelet glycoprotein–specific autoantibodies were detected against GPIIb/IIIa in 18 cases, GPIb/IX in 13 cases. Peripheral blood samples for the N_ITP group were collected from patients with no prior exposure to systemic glucocorticoids. Samples for the C_ITP group were obtained from patients with stable platelet counts and no clinically significant bleeding who were either glucocorticoid-naïve or had discontinued therapy for ≥3 months before sampling. Detailed information was listed in Table S1.

In accordance with the Declaration of Helsinki, the study was approved by the ethics committee of the Second Affiliated Hospital of Wenzhou Medical University (clinical trial number: 2024-K-224-01), and all participants provided written informed consent.

### Method details

#### Data preparation

Control group scRNA-seq data were obtained from GSE175604,^30^ GSE255566.^31^ Raw FASTQ files were processed using CellRanger to generate gene expression matrices, which were subsequently converted into Seurat^41^ objects for downstream analysis. Key steps included quality control, normalization, dimensionality reduction, clustering, and uniform manifold approximation and projection (UMAP) visualization. Cells positive for CD4, CD3E, and protein tyrosine phosphatase, receptor type C (PTPRC) were selected for further analysis (Figure 1B). ChIP-seq data were obtained from GSM8590383^32^ and visualized using the integrative genomics viewer (IGV).^34^ DNA methylation data were downloaded from GSE205495^33^ and processed in R.

#### Single-cell analysis workflow

PCA was used to identify key components, with the top three components visualized. gene ontology (GO) enrichment was performed using the STRING online tool (https://string-db.org/). For scVelo^36^ analysis as shown in Figure 2A, BAM files containing spliced and unspliced RNA information were quality controlled and normalized. RNA velocity was calculated using the scv.tl.velocity() function and projected onto the UMAP for dynamic transcriptional analysis and differentiation trajectory inference. Monocle^42^^,^^43^^,^^44^ was used to reconstruct pseudotime trajectories and identify genes with dynamic expression. Python implementation of Single-Cell rEgulatory Network Inference and Clustering (pySCENIC)^37^ was applied to infer transcription factor regulatory modules by building co-expression networks and integrating motif information to refine gene-regulatory relationships. Regulon activity was computed to assess transcription factor activity across cell states. Cell–cell communication analysis was performed using CellChat.^38^ ComplexHeatmap^39^^,^^40^ was used to draw the heatmap.

#### RT-PCR and western blotting

For reverse transcription polymerase chain reaction (RT-PCR): Total RNA was extracted using TRIzol reagent, with OD260/OD280 ratios above 1.8 as quality control. RNA was reverse transcribed into cDNA, and qPCR was performed with oligo(dT)18 primers, enzyme mix, reaction buffer, DEPC-treated water, and the cDNA template. Relative mRNA levels were calculated using standard formulas. The primers were listed in Table S2. For Western blotting, cells were lysed in RIPA buffer, proteins were quantified, and samples were separated by SDS-PAGE and transferred to PVDF membranes. Membranes were blocked with 5% non-fat milk in TBST, incubated overnight at 4°C with primary antibodies, followed by HRP-conjugated secondary antibodies (Abcam) and visualized via enhanced chemiluminescence. The primary antibodies used in this study included anti-APOO (Thermo Fisher Scientific), anti-CCR7 (Abcam), anti-SP100 (Proteintech), anti-β-actin (Abcam).

#### Flow cytometry and cell sorting

Red blood cells in whole blood samples were lysed using a red blood cell lysis buffer. After centrifugation and washing with PBS containing 2% FBS, the remaining cells were stained with CD4 (BioLegend, 300511), CD8 (BioLegend, 344713), and CD45 (BioLegend, 304011), CCR7 (Biolegend, 353215) antibodies. CD4^+^CD8^−^T cells were gated and sorted via flow cytometry. The data was analyzed by FlowJo v10.10.

#### APOO and pharmacologic modulation

CD4^+^CD8^−^T cells were isolated from peripheral blood of normal donors. Cells were pre-activated for 24 h with anti-CD3/CD28 magnetic beads (1:1 bead-to-cell ratio) and recombinant human IL-2. A total of 1–2 *μ*g plasmid was electroporated into 1×10^6^ pre-activated cells with an optimized electroporation protocol. Post-electroporation, cells were transferred into pre-warmed RPMI-1640 medium supplemented with 10% FBS and IL-2 and incubated at 37°C, 5% CO_2_ for 24–72 h. The efficiency was assessed via qPCR and/or Western blot. To evaluate the impact of mitochondrial metabolism on CCR7 expression, CD4^+^CCR7^+^T cells were cultured and activated under standard conditions and subjected to pharmacological intervention with either the AMPK agonist AICAR or the mitochondrial complex III inhibitor antimycin A. Corresponding vehicle controls (PBS or DMSO) were included in parallel. Following treatment, cells were harvested for quantitative PCR analysis of CCR7 transcript levels and for immunoblotting to assess total protein expression.

#### Structural modeling of TF–DNA interactions

DNA sequences of target genes were retrieved from the national center for biotechnology information (NCBI) database, and amino acid sequences of transcription factors (TFs) were obtained from the AlphaFold Protein Structure Database. These sequences were input into AlphaFold3 server to predict protein-DNA complex structures. The resulting models were visualized and analyzed using PyMOL. Histone modifications and TF binding motifs at gene promoter regions were predicted using the UCSC Genome Browser (https://genome.ucsc.edu/).

#### Seahorse XF analysis

CD4^+^T cells were pre-activated with anti-CD3/CD28 beads and IL-2, then seeded into pre-treated Seahorse XF cell culture plates and incubated overnight. On the assay day, cells were washed and resuspended in assay medium (final volume 500 *μ*L/well). Oligomycin (1 *μ*M), FCCP (2 *μ*M), and rotenone/antimycin A (0.5 *μ*M) were loaded into the probe plate. After calibration, the assay was run on the Seahorse XF analyzer. FCCP concentrations were optimized based on cell type. The Seahorse XF Cell Mito Stress or Glycolysis Stress Test Kits (both from Agilent) were used in this step.

### Quantification and statistical analysis

In this study, data are mostly presented as mean ± standard deviation. For comparison of continuous variables between two groups, *t*-tests were used when data met parametric assumptions; otherwise, non-parametric tests were applied. For the evaluation of the paired experimental design, we employed a paired statistical test. A significance threshold of *p* < 0.05 was used. Correlation analysis was performed using Spearman correlation coefficient. scVelo analyses were conducted on CentOS Stream 9, and all other analyses were performed in R version 4.4.1. The Graphical Abstract and part of workflow were created with BioGDP.com. Graphs were generated using GraphPad Prism.^35^

## References

[1] Al-Samkari H. (2024). 2025 update on clinical trials in immune thrombocytopenia. Am. J. Hematol..

[2] Liu X.G., Hou Y., Hou M. (2023). How we treat primary immune thrombocytopenia in adults. J. Hematol. Oncol..

[3] González-López T.J., Provan D. (2025). The new era of primary immune thrombocytopenia management in adults: A narrative review of current and emerging treatments. Blood Rev..

[4] Sun L., Su Y., Jiao A., Wang X., Zhang B. (2023). T cells in health and disease. Signal Transduct. Target. Ther..

[5] Li Q., Marcoux G., Hu Y., Rebetz J., Guo L., Semple E., Provan D., Xu S., Hou M., Peng J., Semple J.W. (2024). Autoimmune effector mechanisms associated with a defective immunosuppressive axis in immune thrombocytopenia (ITP). Autoimmun. Rev..

[6] Bu S., Liu M., Yang L., Lee P., Miller H., Park C.S., Byazrova M., Filatov A., Benlagha K., Gaber T. (2025). The function of T cells in immune thrombocytopenia. Front. Immunol..

[7] Hou Y., Feng Q., Xu M., Li G.S., Liu X.N., Sheng Z., Zhou H., Ma J., Wei Y., Sun Y.X. (2016). High-dose dexamethasone corrects impaired myeloid-derived suppressor cell function via Ets1 in immune thrombocytopenia. Blood.

[8] Qin J., Liu Q., Liu A., Leng S., Wang S., Li C., Ma J., Peng J., Xu M. (2022). Empagliflozin modulates CD4(+) T-cell differentiation via metabolic reprogramming in immune thrombocytopenia. Br. J. Haematol..

[9] Han L., Zhang L. (2023). CCL21/CCR7 axis as a therapeutic target for autoimmune diseases. Int. Immunopharmacol..

[10] He J., Tsai L.M., Leong Y.A., Hu X., Ma C.S., Chevalier N., Sun X., Vandenberg K., Rockman S., Ding Y. (2013). Circulating precursor CCR7(lo)PD-1(hi) CXCR5^+^ CD4^+^ T cells indicate Tfh cell activity and promote antibody responses upon antigen reexposure. Immunity.

[11] Winter S., Rehm A., Wichner K., Scheel T., Batra A., Siegmund B., Berek C., Lipp M., Höpken U.E. (2011). Manifestation of spontaneous and early autoimmune gastritis in CCR7-deficient mice. Am. J. Pathol..

[12] Zhang L., Zhou G.Z., Feng W.Y., Li D. (2022). Immune Status and Chemokine C Receptor 7 Expression in Primary in Patients with Immune Thrombocytopenia. Turk. J. Haematol..

[13] Guo C., Liu Q., Zong D., Zhang W., Zuo Z., Yu Q., Sha Q., Zhu L., Gao X., Fang J. (2022). Single-cell transcriptome profiling and chromatin accessibility reveal an exhausted regulatory CD4+ T cell subset in systemic lupus erythematosus. Cell Rep..

[14] Chowdhury R.R., D'Addabbo J., Huang X., Veizades S., Sasagawa K., Louis D.M., Cheng P., Sokol J., Jensen A., Tso A. (2022). Human Coronary Plaque T Cells Are Clonal and Cross-React to Virus and Self. Circ. Res..

[15] Aldahlawi A.M., Elshal M.F., Ashgan F.T., Bahlas S. (2015). Chemokine receptors expression on peripheral CD4-lymphocytes in rheumatoid arthritis: Coexpression of CCR7 and CD95 is associated with disease activity. Saudi J. Biol. Sci..

[16] Sen Y., Chunsong H., Baojun H., Linjie Z., Qun L., San J., Qiuping Z., Junyan L., Zhang X., Jinquan T. (2004). Aberration of CCR7 CD8 memory T cells from patients with systemic lupus erythematosus: an inducer of T helper type 2 bias of CD4 T cells. Immunology.

[17] Guo X., Hu J., He G., Chen J., Yang Y., Qin D., Li C., Huang Z., Hu D., Wei C. (2023). Loss of APOO (MIC26) aggravates obesity-related whitening of brown adipose tissue via PPARα-mediated functional interplay between mitochondria and peroxisomes. Metabolism.

[18] Chen J., Hu J., Guo X., Yang Y., Qin D., Tang X., Huang Z., Wang F., Hu D., Peng D., Yu B. (2024). Apolipoprotein O modulates cholesterol metabolism via NRF2/CYB5R3 independent of LDL receptor. Cell Death Dis..

[19] Tang X., Huang Z., Wang F., Chen J., Qin D., Peng D., Yu B. (2023). Macrophage-specific deletion of MIC26 (APOO) mitigates advanced atherosclerosis by increasing efferocytosis. Atherosclerosis.

[20] Barbouche M.R., Chen Q., Carbone M., Ben-Mustapha I., Shums Z., Trifa M., Malinverno F., Bernuzzi F., Zhang H., Agrebi N. (2018). Comprehensive review of autoantibodies in patients with hyper-IgM syndrome. Cell. Mol. Immunol..

[21] Wang C., Zheng X., Jiang P., Tang R., Gong Y., Dai Y., Wang L., Xu P., Sun W., Wang L. (2019). Genome-wide Association Studies of Specific Antinuclear Autoantibody Subphenotypes in Primary Biliary Cholangitis. Hepatology.

[22] Zhao D.T., Yan H.P., Han Y., Zhang W.M., Zhao Y., Liao H.Y. (2023). Prevalence and prognostic significance of main metabolic risk factors in primary biliary cholangitis: a retrospective cohort study of 789 patients. Front. Endocrinol..

[23] Fraschilla I., Jeffrey K.L. (2020). The Speckled Protein (SP) Family: Immunity's Chromatin Readers. Trends Immunol..

[24] Bi E., Ma X., Lu Y., Yang M., Wang Q., Xue G., Qian J., Wang S., Yi Q. (2017). Foxo1 and Foxp1 play opposing roles in regulating the differentiation and antitumor activity of TH9 cells programmed by IL-7. Sci. Signal..

[25] Feng X., Ippolito G.C., Tian L., Wiehagen K., Oh S., Sambandam A., Willen J., Bunte R.M., Maika S.D., Harriss J.V. (2010). Foxp1 is an essential transcriptional regulator for the generation of quiescent naive T cells during thymocyte development. Blood.

[26] Wang H., Geng J., Wen X., Bi E., Kossenkov A.V., Wolf A.I., Tas J., Choi Y.S., Takata H., Day T.J. (2014). The transcription factor Foxp1 is a critical negative regulator of the differentiation of follicular helper T cells. Nat. Immunol..

[27] Choi Y.J., Anders L. (2014). Signaling through cyclin D-dependent kinases. Oncogene.

[28] Shen X., Zhang S., Guo Z., Xing D., Chen W. (2020). The crosstalk of ABCA1 and ANXA1: a potential mechanism for protection against atherosclerosis. Mol. Med..

[29] Araújo T.G., Mota S.T.S., Ferreira H.S.V., Ribeiro M.A., Goulart L.R., Vecchi L. (2021). Annexin A1 as a Regulator of Immune Response in Cancer. Cells.

[30] Luo Y., Xu C., Wang B., Niu Q., Su X., Bai Y., Zhu S., Zhao C., Sun Y., Wang J. (2021). Single-cell transcriptomic analysis reveals disparate effector differentiation pathways in human Treg compartment. Nat. Commun..

[31] Zhao J., Fang Z. (2024). Single-cell RNA sequencing reveals the dysfunctional characteristics of PBMCs in patients with type 2 diabetes mellitus. Front. Immunol..

[32] Jolma A., Hernandez-Corchado A., Yang A.W.H., Fathi A., Laverty K.U., Brechalov A., Razavi R., Albu M., Zheng H., Codebook Consortium (2024). GHT-SELEX demonstrates unexpectedly high intrinsic sequence specificity and complex DNA binding of many human transcription factors. bioRxiv.

[33] Du H., Tang Q., Yang J., Yan B., Yang L., Wang M. (2023). Genome-wide DNA methylation profiling of CD4+ T lymphocytes identifies differentially methylated loci associated with adult primary refractory immune thrombocytopenia. BMC Med. Genomics.

[34] Robinson J.T., Thorvaldsdóttir H., Winckler W., Guttman M., Lander E.S., Getz G., Mesirov J.P. (2011). Integrative genomics viewer. Nat. Biotechnol..

[35] Jiang S., Li H., Zhang L., Mu W., Zhang Y., Chen T., Wu J., Tang H., Zheng S., Liu Y. (2025). Generic Diagramming Platform (GDP): a comprehensive database of high-quality biomedical graphics. Nucleic Acids Res..

[36] Bergen V., Lange M., Peidli S., Wolf F.A., Theis F.J. (2020). Generalizing RNA velocity to transient cell states through dynamical modeling. Nat. Biotechnol..

[37] Van de Sande B., Flerin C., Davie K., De Waegeneer M., Hulselmans G., Aibar S., Seurinck R., Saelens W., Cannoodt R., Rouchon Q. (2020). A scalable SCENIC workflow for single-cell gene regulatory network analysis. Nat. Protoc..

[38] Jin S., Plikus M.V., Nie Q. (2025). CellChat for systematic analysis of cell-cell communication from single-cell transcriptomics. Nat. Protoc..

[39] Gu Z., Eils R., Schlesner M. (2016). Complex heatmaps reveal patterns and correlations in multidimensional genomic data. Bioinformatics.

[40] Gu Z. (2022). Complex heatmap visualization. iMeta.

[41] Hao Y., Stuart T., Kowalski M.H., Choudhary S., Hoffman P., Hartman A., Srivastava A., Molla G., Madad S., Fernandez-Granda C., Satija R. (2024). Dictionary learning for integrative, multimodal and scalable single-cell analysis. Nat. Biotechnol..

[42] Trapnell C., Cacchiarelli D., Grimsby J., Pokharel P., Li S., Morse M., Lennon N.J., Livak K.J., Mikkelsen T.S., Rinn J.L. (2014). The dynamics and regulators of cell fate decisions are revealed by pseudotemporal ordering of single cells. Nat. Biotechnol..

[43] Qiu X., Mao Q., Tang Y., Wang L., Chawla R., Pliner H.A., Trapnell C. (2017). Reversed graph embedding resolves complex single-cell trajectories. Nat. Methods.

[44] Qiu X., Hill A., Packer J., Lin D., Ma Y.A., Trapnell C. (2017). Single-cell mRNA quantification and differential analysis with Census. Nat. Methods.

