# Supplementary material for: Dysregulation of apolipoprotein o reprograms CCR7+CD4+T cell fate in primary autoimmune thrombocytopenia
Source: iScience. 2025 Oct 16;28(11):113792. doi: 10.1016/j.isci.2025.113792 (PMC12616023; doi:10.1016/j.isci.2025.113792)
Supplement: Document S1. Figure S1 and Tables S1 and S2 [file mmc1.pdf]

iScience, Volume *28*

Supplemental information

Dysregulation of apolipoprotein o reprograms CCR7^{+}CD4^{+}T cell fate in primary autoimmune thrombocytopenia

Tengda Li, Xiang Li, and He Huang

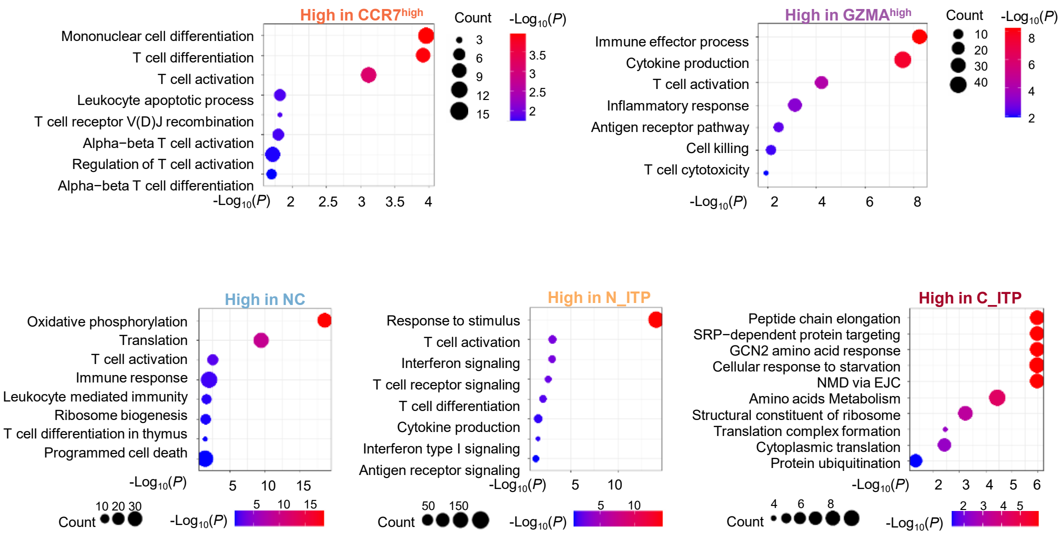

Figure S1. The pathway enrichment of high expressed genes in different subsets, related to Figure 1.

Number	Normal		Patient		Anti-platelet antibodies		Disease stages			Samples were collected before therapy	Samples were collected for patients without treatment for more than 3 months	Current treatment regimens	
	Age(Year)	Gender	Age(Year)	Gender	GPIIb/IIIa(+)	GPIb/IX(+)	Newly diagnosed	Chronic	Refractory			Glucocorticoid	Intravenous immunoglobulin
1	50	Female	48	Female	✓	×	✓	×	×	✓	×	✓	✓
2	49	Female	50	Female	✓	×	✓	×	×	✓	×	✓	×
3	51	Female	50	Female	✓	×	✓	×	×	✓	×	✓	×
4	51	Female	51	Female	✓	×	✓	×	×	✓	×	×	×
5	53	Female	51	Female	✓	×	✓	×	×	✓	×	×	×
6	53	Male	51	Female	×	✓	✓	×	×	✓	×	×	×
7	54	Male	52	Male	✓	×	✓	×	×	✓	×	×	×
8	55	Male	53	Male	✓	×	✓	×	×	✓	×	×	×
9	56	Male	52	Male	✓	✓	✓	×	×	✓	×	×	×
10	51	Male	53	Male	✓	✓	✓	×	×	✓	×	×	×
11	52	Female	53	Male	✓	✓	✓	×	×	✓	×	×	×
12	52	Female	50	Male	✓	✓	✓	×	×	✓	×	×	×
13	53	Female	50	Female	✓	×	×	✓	×	✓	×	✓	×
14	54	Female	51	Female	✓	×	×	✓	×	✓	×	✓	×
15	49	Female	51	Female	✓	×	×	✓	×	×	✓	✓	×
16	50	Male	52	Female	✓	×	×	✓	×	×	✓	✓	×
17	52	Male	52	Female	✓	✓	×	✓	×	×	✓	✓	✓
18	53	Male	52	Female	×	✓	×	✓	×	×	✓	✓	✓
19	51	Male	53	Male	✓	✓	×	✓	×	×	✓	✓	✓
20	53	Male	53	Male	✓	✓	×	✓	×	✓	×	✓	✓
21			54	Male	×	✓	×	✓	×	✓	×	✓	✓
22			51	Male	×	✓	×	✓	×	✓	×	✓	×
23			48	Male	×	✓	×	✓	×	✓	×	✓	×
24			55	Male	×	✓	×	✓	×	✓	×	✓	×

Table S1.Clinical information of the enrolled participants, related to Figures 1-4.

Genes	Forward (5' → 3')	Reverse(5' → 3')
APOO	taattcagaggtccgtgggg	gcaatagtgtcggagctgtg
CCR7	cttcggtgtccacttttgca	cttgctgatgagaaggacgc
CDK6	tgtttcagcttctccgaggt	tatgcagccaacactccaga
SP100	gctaagtatacgctgcggtg	cccatttgttgcacacctca
FOXP1	cagcaacagtggcatctcat	aagggttaggctgacacaca
LDLRAP1	ttcagcctcaagtacctggg	ggtgaggttgtctgtcagga
MT-ATP8	accgtatggcccaccataattacc	tttatgggctttggtgagggaggt
MT-ND4L	tatcgctcacacctcatatcctccct	aggcggcaaagactagtatggcaa
PTPRCAP	aatgaccttgagcgacagga	gtcactgtctctggcttcct
ANXA1	atgcacagcgtcaacagatc	tcagttccaaggcccttcat

Table S2.Primers used in this study, related to Figure 4.
